# A novel noble metal stent coating reduces in vitro platelet activation and acute in vivo thrombosis formation: a blinded study

**DOI:** 10.1038/s41598-023-44364-4

**Published:** 2023-10-11

**Authors:** Mikael Sandell, Anna Ericsson, Jonathan Al-Saadi, Billy Södervall, Erika Södergren, Stefan Grass, Javier Sanchez, Staffan Holmin

**Affiliations:** 1 Division of Micro and Nanosystems, KTH Royal Institute of Technology, Malvinas väg 10, 114 28 Stockholm, Sweden; 2 Department of Clinical Neuroscience, Karolinska Institute, Tomtebodavägen 18A, 171 77 Stockholm, Sweden; 3 MedTechLabs, Stockholm, Sweden; 4 Bactiguard AB, Alfred Nobels allé 150, 146 48 Tullinge, Sweden; 5 Department of Clinical Sciences, Danderyd Hospital, 182 88 Stockholm, Sweden

**Keywords:** Biomedical materials, Preclinical research, Acute inflammation, Thrombosis, Platelets, Blood flow, Nanostructures

## Abstract

Inherent to any stenting procedure is the prescription of dual antiplatelet therapy (DAPT) to reduce the platelet response. Clinical guidelines recommend 6–12 months of DAPT, depending on stent type, clinical picture and patient factors. Our hypothesis is that a nanostructured noble metal coating has the potential to reduce protein deposition and platelet activation. These effects would reduce subsequent thrombo-inflammatory reactions, potentially mitigating the need for an extensive DAPT in the acute phase. Here, a noble metal nanostructure coating on stents is investigated. Twelve pigs underwent endovascular implantation of coated and non-coated stents for paired comparisons in a blinded study design. The non-coated control stent was placed at the contralateral corresponding artery. Volumetric analysis of angiographic data, performed by a treatment blinded assessor, demonstrated a significant thrombus reduction for one of the coatings compared to control. This effect was already seen one hour after implantation. This finding was supported by in vitro data showing a significant reduction of coagulation activation in the coated group. This novel coating shows promise as an implant material addition and could potentially decrease the need for DAPT in the early phases of stent implementation.

## Introduction

The placement of stents for the prevention of atherosclerotic stenosis and the treatment of dissections and aneurysms at different sites of the vascular system is increasingly important. Several properties of stents, such as radiopacity, length, expanded size, surface topography, charge, hydrophobic/hydrophilic interactions, or surface area, play an important role in long-term outcomes; however, above all properties, the type of material used for their construction, design, and coating has a significant influence^1^. Stent implantation into the vasculature immediately initiates rapid protein adsorption on the surface that induces the activation of coagulation, platelets, and leucocytes, possibly causing adverse effects such as acute thromboembolic complications, bleeding, or pro-inflammatory reactions^2,3^. This pathophysiology has been studied in detail and is known as the thrombo-inflammatory reaction^4^. Once platelets are activated, intracellular granules are released, mainly promoting the amplification of platelet activation, blood coagulation, and inflammation, causing either acute stent thrombosis (ST) or in-stent restenosis (ISR). This problem is present on any vascular implant and necessitates the administration of dual antiplatelet therapy (DAPT), which could be administered for up to a year^5,6^. This treatment, while preventing acute and long-term complications such as ST, poses an increased risk of bleeding in the short and longer term^7^. For patients requiring additional surgical interventions, this is especially troublesome. If the thrombo-inflammatory reaction could be reduced in the acute phase the cascade reaction associated with platelet activation as well as other associated physiological reactions will be reduced. Provided these benefits, the DAPT could potentially be reduced to single medication treatment or potentially no treatment during the early post-stent implantation phase, further benefitting patients.

Attempts to target the thrombo-inflammatory reaction with various surface modifications have been made. A review of implant material surface interactions with blood describes two types of surface modifications, passive and bioactive^8^. Bioactive surface modifications, such as drug-eluting stents (DES), target selective parts of the cellular interactions with the surface, either inhibiting or promoting them^9,10^. Passive modifications are instead aimed at preventing non-specific protein binding on the surface, hence reducing the subsequent foreign body response. Adsorbed fibrinogen is identified as the main factor in platelet adhesion and activation, and in some instances, it is argued that the role of a successful surface modification lies in the nature of the conformational change of the adsorbed proteins^11^. We hypothesize that a surface coating that reduces protein adsorption, specifically the formation of fibrin, could potentially mitigate acute thrombosis. Noble metals, and alloys thereof, have been used as surface coatings due to good corrosion resistance as well as improved blood compatibility^12–14^. The technology developed by Bactiguard™ (Stockholm, Sweden) to coat medical devices consists of a nanostructured layer with a combination of a noble metal alloy (NMA), modifying the surface and reducing the adhesion of microbes, therefore reducing the risk of microbial infections^15,16^. This type of coating also presents a high degree of biocompatibility, which has been investigated in clinical trials^17,18^.

The present study evaluates a modified version of this NMA coating, containing neodymium (Nd), when used on nitinol stents, tubes or plates. We developed a methodology where the coating performance compared to the control material is investigated in vivo in the acute phase, with 3D angiographic evaluation and volumetric analysis, as well as in vitro using a chandler loop model (CHL). We compared the different cascades of activation of the defense mechanisms in blood in comparison with the uncoated control material. The coating significantly reduced ST in the acute phase in vivo and also significantly lowered the in vitro activation of coagulation and fibrin deposition.

## Methods

### Material preparation

The EverFlex 5 × 40 mm Peripheral Self-Expanding Stent System (Medtronic, MN, USA) was used throughout the in vivo porcine experiments. Prior to the coating procedure, the stents were removed from the delivery catheter. The stents were coated in a wet chemical process carried out at room temperature where the substrate to be coated is dipped in a sequence of water-based baths containing the ions of the different metals. The coating is made up of a mixture of noble metals, which are firmly immobilized in a stochastic distribution without being released during use. The obtained coating has an average layer thickness less than 2 nm. After the coating the stents are reinserted into the delivery catheter. The reinsertion procedure was realized using an HV500 Disposable Heart Valve Crimping Tool (Machine Solutions Inc., AZ, USA) and consisted of crimping the stent, positioning it at the end of the aperture, aligning it with the opening of the original delivery catheter, and pushing it in with a delivery rod from another EverFlex catheter system. The stents in the control group were also removed from the delivery catheter and reinserted to ensure similar treatment between groups, but not coated.

Nitinol tubes and plates were used in the in vitro porcine and human experiments. These were coated in the same batch as the stents and subjected to CHL experiments (tubes) and contact angle measurements (plates).

### Experimental design

**Figure 1:**
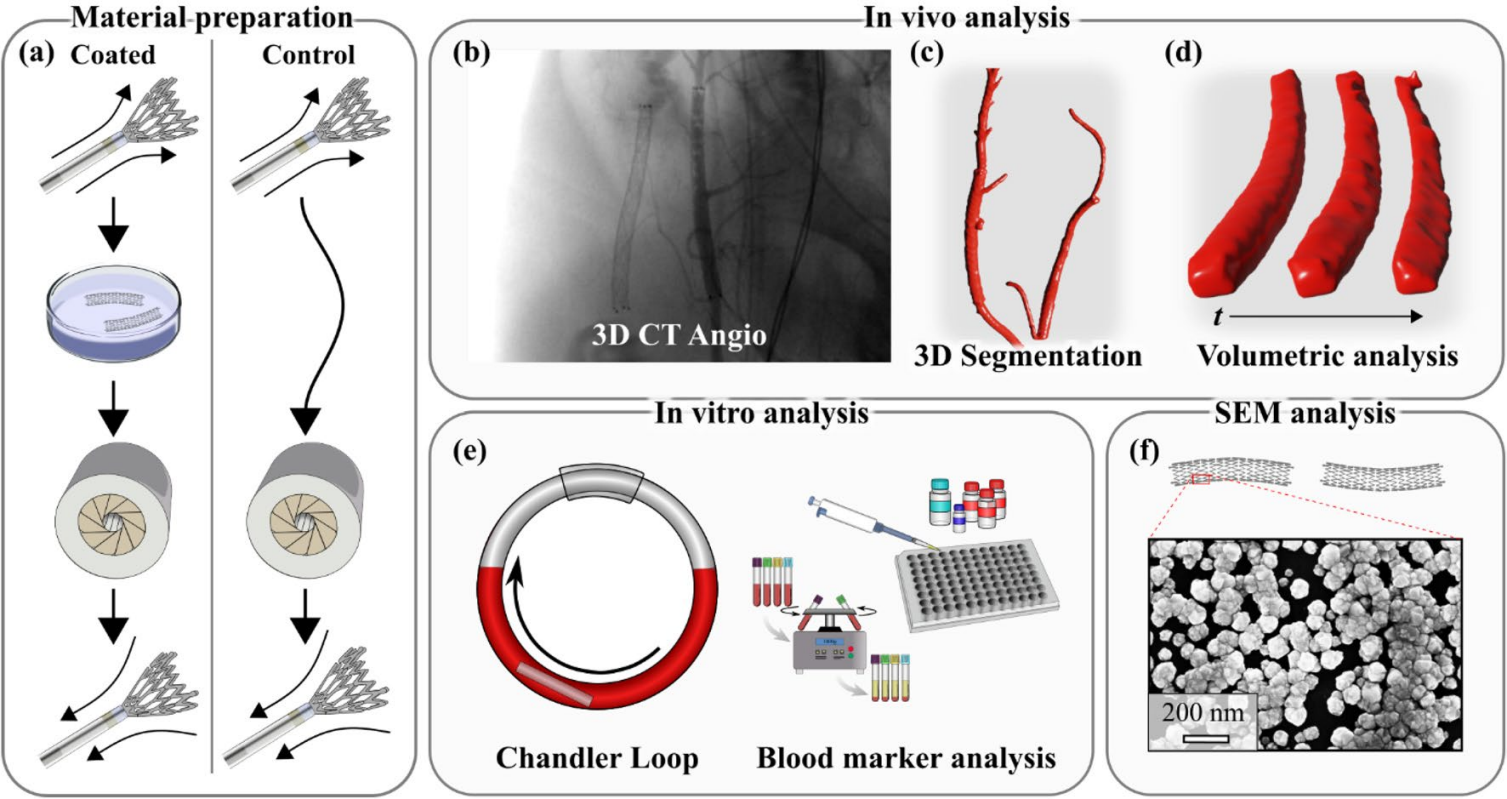
Experimental procedure of the current study. Material preparation in the form of stent extraction, coating and reinsertion (**a**). In vivo analysis consisting of 3D CT angio scans (**b**), coupled with 3D segmentation (**c**) and volumetric analysis over time (**d**). In vitro experiments utilizing blood sampling and chandler loop models coupled with ELISA and Multiplate analysis kits for blood marker analysis (**e**). Analysis of the coatings integrity after implantation with SEM (**f**).

The study was performed as a parallel, blinded in vivo and in vitro study. In the in vivo porcine experiments, coated and uncoated stents were implanted pairwise in each animal and monitored with angiographic scans over time, comparing their performance. Additionally, blood was extracted for Chandler loop experiments, and at various time points in the in vivo experiments to measure the activation of the coagulation system, Table 1. The investigation was two-pronged; one part focused on the acute thrombosis formation in vivo, and the other focused on the effect of the coating on blood markers associated with thromboembolic activation (Fig. 1). The blinded setup was designed so that neither the surgeon nor the head of the investigation knew which stent or nitinol tube belonged to which group.

**Table 1:** The layout of parallel in vivo and in vitro experiments.

Event	In vivo experiment	In vitro experiment
Animal arrives	Blood extracted for CHL and sample P1	CHL start
Catheterization complete	Blood sample P2	
Implantation of 1st stent	3D angio & blood sample P3	
Implantation of 2nd stent	3D angio & blood sample P4	
1 h after 1st stent	3D angio & blood sample P5	
1 h after 2nd stent	3D angio	
2 h after 1st stent	3D angio & blood sample P6	
2 h after 2nd stent	3D angio	CHL end
Euthanasia	Stent explantation	

CHL, chandler loop.

#### In vivo evaluation

Angiographic evaluation of the stent prototypes was performed in animal experiments, in a total of 12 naïve, adult, mixed-gender, Yorkshire-Swedish farm swine, weighing 39.3 ± 2.0 kg. Animals were pre-medicated with sedatives (intramuscular injection of tiletamine [2.5 mg kg^−1^], zolazepam [2.5 mgkg^−1^], and medetomidine [0.1 mg kg^−1^]) and taken to a fully equipped clinical angiography suite, intubated and mechanically ventilated while receiving standard surgical anesthetic care. Anesthesia was induced with intravenous (i.v.) propofol (20 mg) or i.v. sodium pentobarbital (120–180 mg) and maintained with sodium pentobarbital infusion (15–20 mg kg^−1^ h^−1^). Analgesia was achieved using i.v. fentanyl (50 µg h^−1^). Throughout the intervention, the animals were monitored for complications using electrocardiogram, invasive blood pressure measurement, oxygen saturation, temperature, and urine production. Immediately after each stent implantation, the animals were given local injections of Nimotope (0.25 mg) via the guide catheter to alleviate vasospasms during the implantation procedures. At the end of the experiment, animals were sacrificed using a lethal dose of sodium pentobarbital (100 mg kg^−1^). The stents were explanted and photographed (Supplementary Fig. 1).

Stent implantations (*n* = 24) were performed pairwise; a coated (*n* = 12) and uncoated (*n* = 12) stent implanted in each animal in the left and right branch of the lingual artery, respectively. The lingual artery was chosen due to identical endovascular approach to the left- and right-hand side and ease of post-mortem dissection of the stents. Sample size was selected based on the resource equation method^19^. The stents were evaluated with 3D angiographies with contrast injections bilaterally in the distal common carotid artery at three different time points: directly after stent implantation, one hour after implantation, and two hours after implantation. The angiographies were performed with a syringe pump (MedRad ProVis Mark V) with an injection rate of 20 ml s^−1^ for 4 s. Blood samples were obtained at various timepoints to investigate the effect on the coagulation system of the implantation, and endovascular navigation.

#### 3D angiography analysis

The open-source software ITK-SNAP (Penn Image Computing and Science Laboratory, USA) was used to measure the contrast volume that fills the stent at different time points^20^. Digital Imaging and Communications in Medicine (DICOM) datasets, generated from Angio CT scans, were imported into the software, and a semi-automatic active contour segmentation was performed. After the segmentation procedure, the region of interest (ROI) was further refined manually by removing coronal segments that continued beyond the end of the stents, as indicated by the four radio markers, discontinuous voxels, and voxels that entered vessels branching out from the stent. The volume of the ROI is then compiled in the software in terms of a voxel count, which is the value that was used in subsequent analyses. To remove size effects in the analysis the volumes were normalized based on the volume at the time of delivery, by dividing the voxel count of follow-up stent volumes with the voxel count at delivery. The stent-artery ratio was measured across a subset of experiments (*n* = 10). All measurements were blinded. Exclusion criteria during 3D analysis were extensive unilateral vasospasm or vessel dissection.

#### In vitro evaluation

A modified CHL was used to assess blood compatibility of the noble metal coating materials as previously described^4,12,21^. In the CHL experiments coated and uncoated nitinol tubes were used and compared to each other. An empty loop was included to compare with the spontaneous blood activation connected to the experiment itself. Additionally, unexposed blood from each CHL experiment was analyzed in order to establish baseline values. The nitinol tubes were 3 cm in length with a 3 mm outer diameter. The ends of the tubes were sealed with polyurethane in order to avoid unwanted turbulent flows inside the tube. The loops were 30 cm in length, oriented with a 9.55 cm bending radius, with a 6.5 mm internal diameter of polyvinyl chloride (PVC) tubes (Medtronic, Minneapolis, USA) internally coated with immobilized heparin. CHL experiments were performed with porcine blood (*n* = 12), and human blood (*n* = 20). The porcine and human CHL experiments were performed pairwise with the coating versus the control group. The CHL experiments with porcine blood were performed in parallel with the in vivo experiments and the CHL experiments with human blood were performed separately.

In all CHL experiments, the tubing was initially pre-treated with saline for 5 min after which the nitinol tubes were placed inside the tubes. The loop was then filled with blood, closed using a connector with the same internal coating specification, mounted on a Plexiglas plate, and rotated vertically with 10 rpm. In the porcine blood the experiment was performed in 2 h, in human blood it was performed in 1 h. The blood was then removed from the loops and was transferred into an EDTA (BD VacutainerVR, K2E [5.4 mg]), a citrate (BD VacutainerVR, 9NC [0.129 M]) and a Hirudin (SARSTEDT, S-Monovette Hirudin [> 525 ATU mL^−1^]) tube with the purpose to stop subsequent reactions. The EDTA and citrated tubes containing blood were centrifuged, and plasma was collected and stored at − 80 °C until analysis.

Throughout the in vivo experiments, blood samples were taken at various time points to evaluate the systemic influence of catheterization and implantation of each stent on several blood biomarkers. Blood analysis was performed to check for correlating blood markers and notable differences between groups. The blood compatibility analysis was carried out according to ISO 10993:4, which includes analysis and evaluation of the coagulation, platelets, and leucocytes. The thrombin-antithrombin (TAT) level formation, polymorphonuclear elastase (PMN), and beta-thromboglobulin (*β*-TG) analysis was performed using ELISA kits according to manufacturer instruction. Adenosine diphosphate (ADP) and thrombin receptor activating peptide (TRAP) were measured with a Multiplate Analyzer (Roche, Basel, Switzerland) carried out according to instruction by the manufacturer. Maximum platelet aggregation and aggregation velocity are expressed and presented in arbitrary units (Area under curve [U]).

After blood incubation, the exposed tubes were washed using saline and stained with hematoxylin (0.1%) for 30 min. The fibrin deposition on the tubes was photographed and evaluated using ImageJ in order to assess the degree of fibrin coverage formed during the exposure (Supplementary Fig. 1).

### Ethical considerations

All research was conducted in accordance with national and local guidelines for Sweden and Karolinska Institutet, respectively. All animal experiments had ethical approval from the local ethics committee (Stockholms Norra Djurförsöksetiska Nämnd, Stockholm, Sweden, Dnr 525-2018 & 13170-2020) and all methods were performed in accordance with 'Principles of Laboratory Animal Care' formulated by the National Society for Medical Research and the 'Guide for the Care and Use of Laboratory Animals' prepared by the Institute of Laboratory Animal Resources and published by the National Institutes of Health. The in vivo experiments in this study is reported in accordance with the ARRIVE guidelines. The research on human blood was approved by the National Ethics Committee at Karolinska Institute in Stockholm, Sweden (Dnr 2010/1627-31/3), and informed consent was given by all blood donor participants.

### Coating and surface characterization

The coating on both the stents and the nitinol tubes used in the CHL experiments was examined with SEM (Zeiss Ultra 55) before and after the experiments to evaluate coating integrity. Contact angle measurements were performed with the Sessile drop method on an optical tensiometer (OneAttension, Biolin Scientific) on flat nitinol plates, subjected to the same coating procedure, with 10 μl droplets of deionized water (DIW) (*n* = 8), human blood plasma (*n* = 8), and human whole blood (*n* = 8).

### Statistical analysis

The statistical evaluation was done in GraphPad Prism (v9.1.2). Descriptive statistics are presented as mean ± SD unless otherwise stated. For the in vivo volume measurements, a two-tailed, paired *t*-test was applied for each time point, comparing the mean of each group with its respective control group. For the in vitro human experiments, a two-tailed, paired *t*-test was applied. For the porcine experiments, the data points are not paired, thus a Mann Whitney *t*-test was performed across all groups to determine the significance level. The significance level for all tests was set at *p* < 0.05. Levels of significance are indicated with a number of asterisks (*) dependent on the *p*-value range and non-significant measurements are indicated with ns, according to the GraphPad style of significance levels^22^.

## Results

### In vivo volumetric analysis

**Figure 2:**
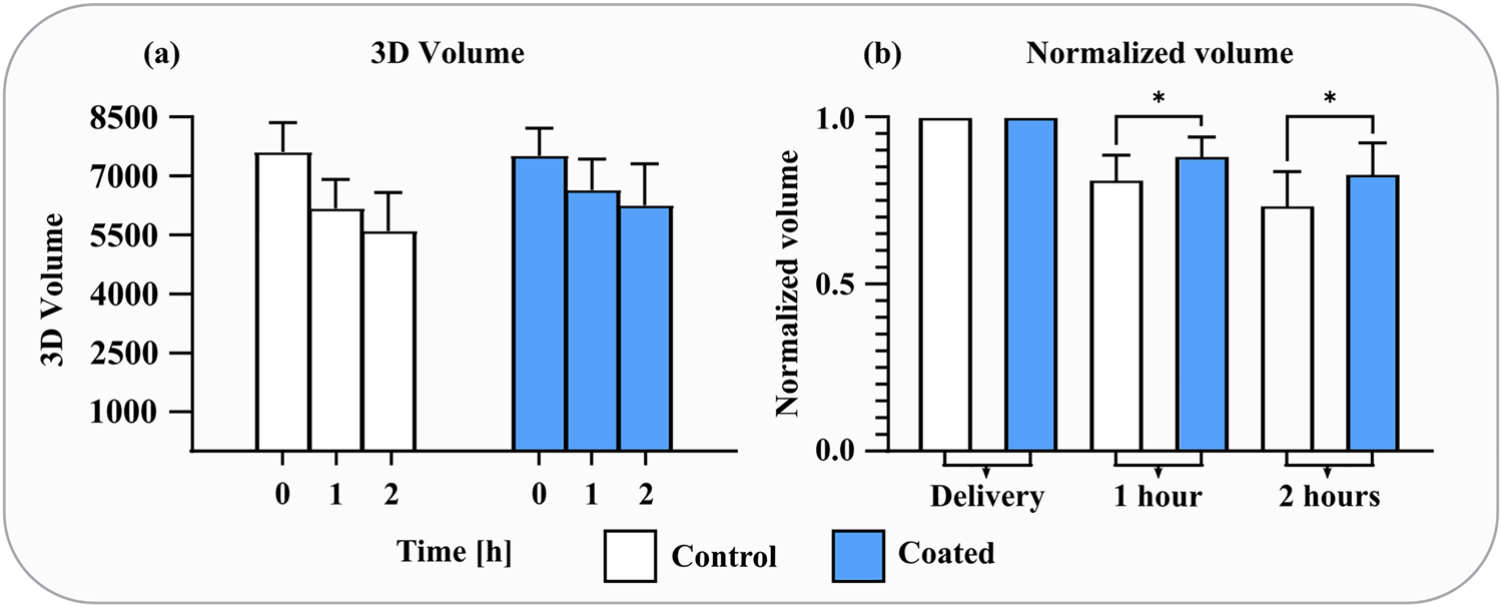
Results from animal trials (n = 9) of the volumetric analysis for the control stents (white) and coated stents (blue). Data for the absolute voxel volumes of contrast-filled lumen (**a**) generated from ITK-Snap and the normalized volumes (**b**) that account for differences in vessel size. There was a significant improvement in the coated group compared to the control group with normalized vessel volumes.

Analysis of the angiography data consisted of measuring the 3D volume of the contrast pillar that fills the stents. A volume decrease is linked to the formation of a thrombus following platelet activation, which reduces the contrast-filled vessel lumen. A comparison was made between the control group and coated group in terms of absolute volume and normalized volume based on the initial time point. Due to extensive unilateral vasospasm, data from three animal trials were excluded. The stent-artery ratio across both groups was 1.37 ± 0.11 and was not significantly different between the control and coated group. The analysis revealed a statistically significant difference between the coated group and the control group regarding acute stent thrombosis (ST) when volumes were normalized (Fig. 2). A significant improvement was revealed both at the 1-h (*p* = 0.0476) and 2-h mark (*p* = 0.0405).

### Blood marker analysis

**Figure 3:**
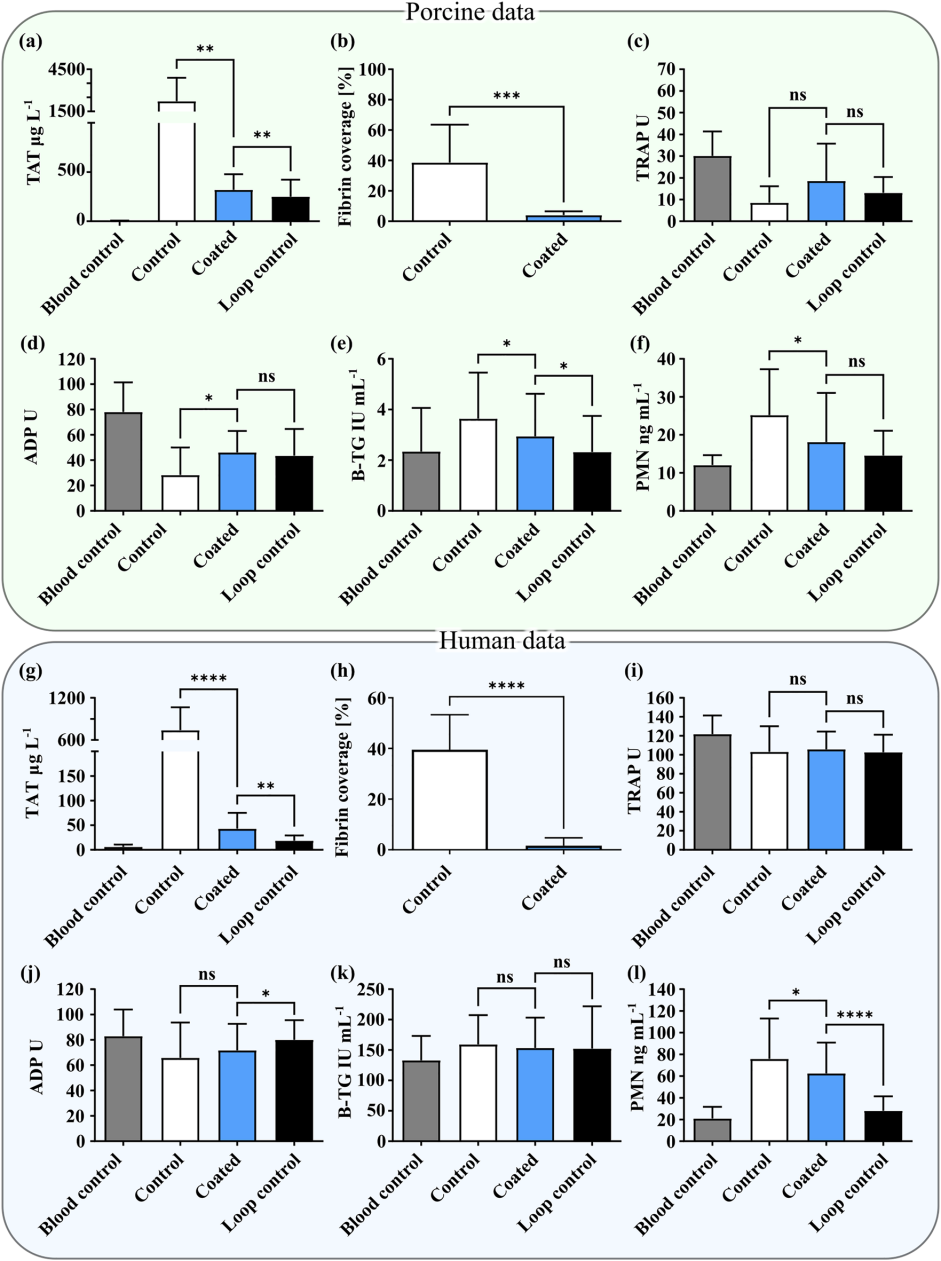
In vitro data and statistical analysis from the CHL experiments, divided into porcine (n = 12) (**a**–**f**) and human (n = 20) (**g**–**l**) data. TAT and fibrin generation show a clear reduction in the coated group in both experiment modalities. The coated group shows a significant difference in porcine blood for all markers except TRAP. In human blood, the corresponding measurements show a significant improvement for PMN.

The results from the in vitro CHL show that the coated material reduces the coagulation activation system as compared to the control material when exposed to both human and porcine blood for 1 and 2 h, respectively (Fig. 3a,b and g,h). Data from the in vitro experiments are presented as a comparison of baseline levels (Blood control), measurements from the coated and control material, as well as measurements from the spontaneous activation from the empty loops (Loop control). Several notable differences between the groups were revealed. In the TAT level measurements, there was a distinct decrease in the coated group compared to control (*p* < 0.01). The corresponding measurement in human blood showed an even larger decrease in the coated group (*p* < 0.0001). Another important evaluation parameter is the deposition of proteins on the surfaces, especially fibrin deposition. The difference in fibrin coverage between the coated group and control was clearly demonstrated in the porcine data (*p* < 0.001). The corresponding effect in human blood showed a similar difference between the coated and control groups for the fibrin coverage measurement (*p* < 0.0001). The coated group showed improvements compared to the control group for ADP, *β*-TG and PMN in porcine blood (*p* < 0.05) and for PMN in human blood (*p* < 0.05) (Fig. 3d–f and l). In the remaining measurements, there was no significant difference between the coated group or control group (*p* > 0.05) (Fig. 3c and i–k). All markers measured in vivo indicated no significant difference between the groups (Supplementary Table 1). The measurement of platelet consumption in the human experiments showed a significant difference between the coated group and control group (*p* < 0.0001), with a lower platelet consumption in the coated group (Table 2). Additionally, there was a significant difference for the C3a-marker between the coated group and control group (*p* < 0.05). Furthermore, there was no significant difference between the coated group and the spontaneous activation measured in the loop control (*p* > 0.05).

**Table 2:** Platelet count and complement activation markers after human CHL.

	Loop control	Coated	Control	Significance
Platelet count (%)	91.4 ± 8.3	90.0 ± 5.7	73.1 ± 6.4	****
Complement activation, C3a (ng ml^−1^)	554.2 ± 167.0	651.3 ± 134.9	822.1 ± 352.0	*

**Figure 4:**
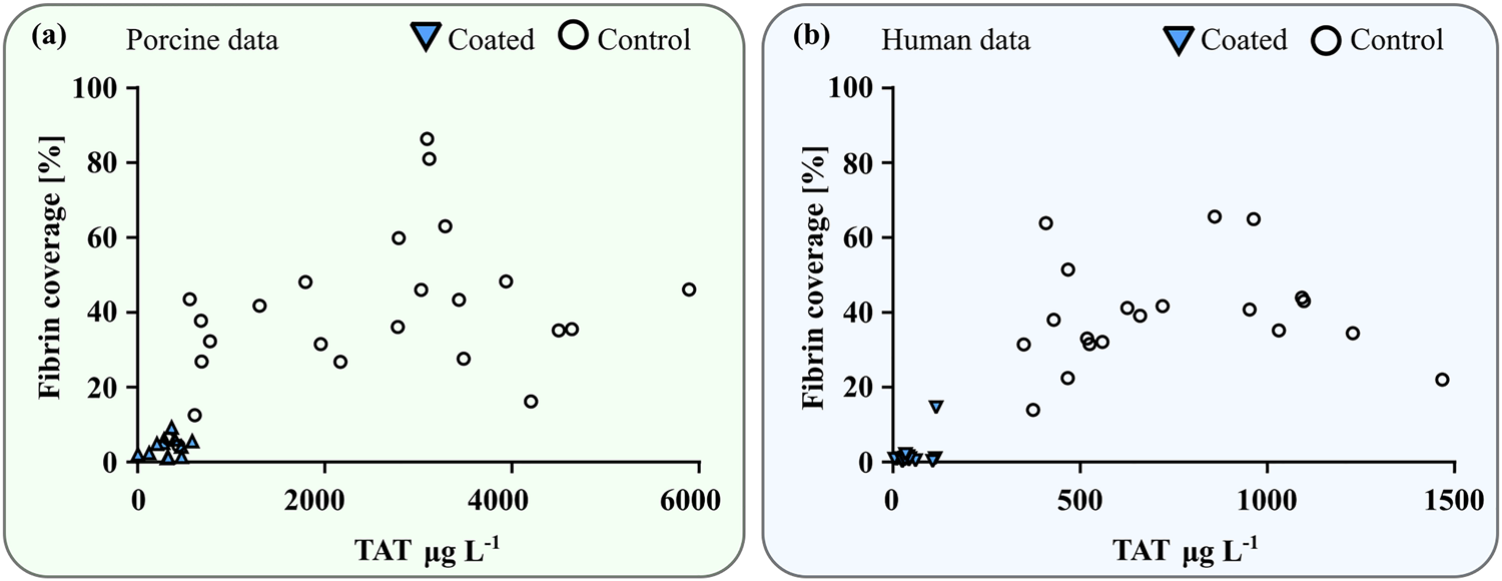
TAT and fibrin scatter plots for porcine experiments (**a**) and human experiments (**b**). The coated group shows a substantial reduction in both measured quantities.

A correlation was observed between high levels of TAT generation and increased fibrin deposition on the tubes exposed to both pig and human blood. The lower TAT values in the coated groups showed minimal fibrin coverage (Fig. 4). Significance based on paired *t*-test of the coated and control group (*n* = 20).

### Coating characteristics

**Figure 5:**
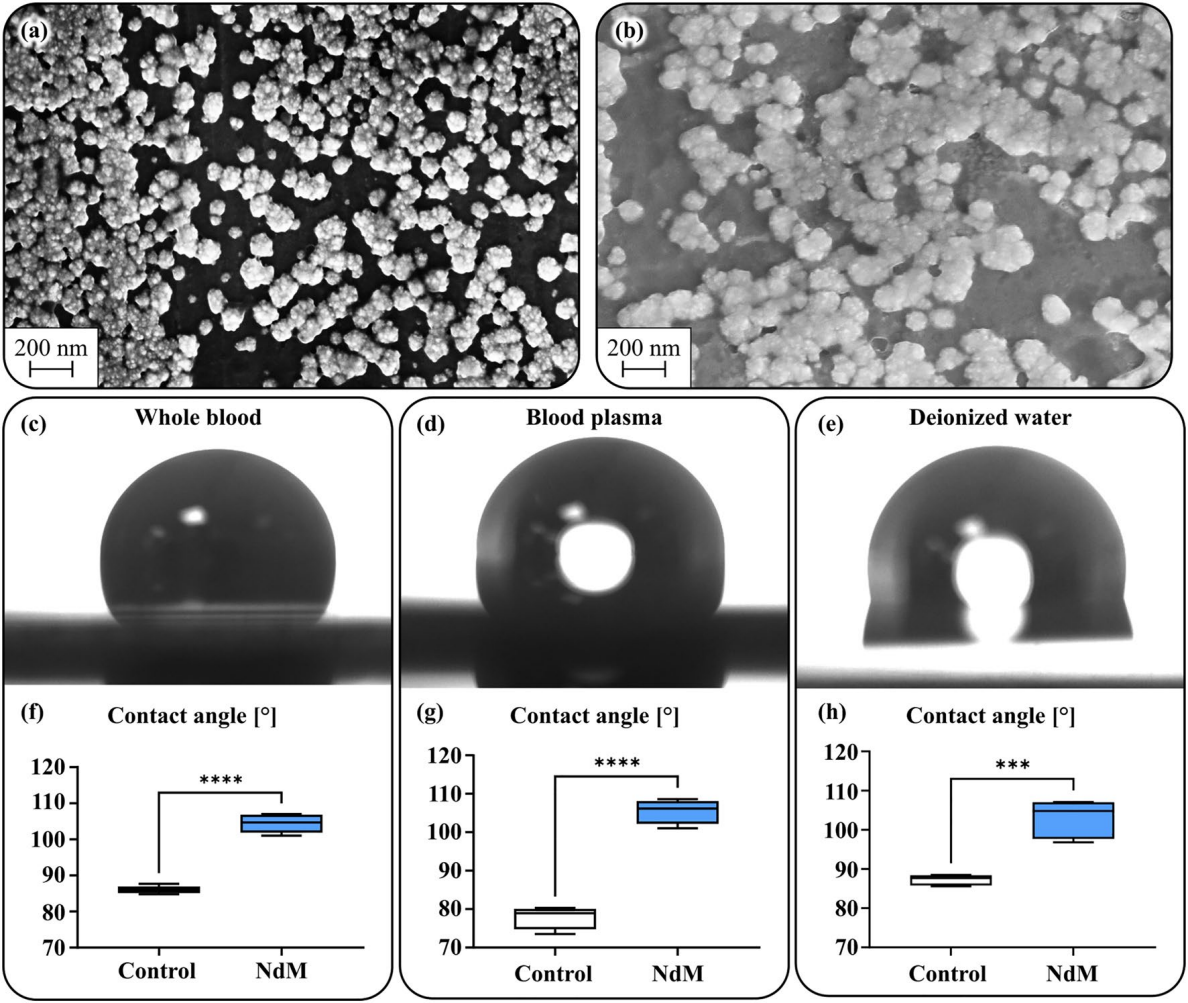
SEM images of the coated surface prior to implantation (**a**) and after implantation (**b**). Camera images of individual contact angle measurements of whole blood, blood plasma and DI water (**c**–**e**) and statistical analysis of the measurements (**f**–**h**). Significant differences are observed across all measurements.

The stent and tube surfaces were investigated in a scanning electron microscope (SEM) prior to and after the in vivo and in vitro experiment. The coating was not notably affected by either experimental setup (Fig. 5a,b). The contact angle was measured for three liquids: whole blood, blood plasma, and deionized water (DIW). Increased contact angles in the coated group were observed across all measurements (Fig. 5c–h).

## Discussion

The novel coating investigated here significantly reduces acute in vivo stent thrombosis and reduces in vitro coagulation activation compared to control. This effect is seen already after 2 h in vitro and 1 h in vivo. The replacement of Ag with a lanthanide component, Nd, in a well-known infection prevention coating^16,23,24^ was done to have a better performance for thrombosis prevention than the standard coating, due to demonstrated anticoagulant effects of Nd^25^.

Despite the significant advances in stent development, severe complications such as acute ST still occur^26^. To prevent this, DAPT is given to all patients undergoing stent implantation. DAPT has clear negative medium- and short-term effects, and the need for additional surgical procedures during the acute phase are riskier under DAPT. In clinical trials, the degree of platelet activation from stent implantation has been studied in both the short and long term^27,28^. Studies carried out with different types of materials and designs have provided data on the degree of thrombogenicity of stents, showing that the longer the stents, the greater the platelet activity^29^. New endovascular products, like flow-diverters for aneurysm treatment with higher density metal mesh, also highlights the need for new strategies to prevent acute ST. Alternative pharmacological strategies have also been tested to reduce the recurrence of acute ST, such as the combined use of different drugs, e.g. heparin, aspirin, and ticlopidine^30,31^. An alternative method for reducing the DAPT duration, such as a modification of the stent surface, would be of great help when handling patients given a stent implant. Surface modifications can be tailored to target different aspects of the foreign body response, such as protein adsorption, platelet aggregation and the inflammatory reactions^8^.

The rapid absorption of plasma proteins on the surface of stents initiates the process of activation of thrombus formation. This protein absorption is reversible and dynamic, changing its composition during the exposure time; a phenomenon known as the Vroman effect^32^. This phenomenon is directly related to negatively-charged hydrophilic surfaces. The first proteins to colonize the surface are fibrinogen and the contact activation system proteins, including factor XII (FXII), factor XI (FXI), prekallikrein, and high molecular weight kininogen, which trigger coagulation when activated^33^. Thrombin is then generated, converting fibrinogen into fibrin. This process ends up amplifying the activation of coagulation, attracting, and activating, platelets and leukocytes, which induces the activation of the inflammatory system^34,35^. Here, we present a nanostructured noble metal surface coating applied on stents, aimed at reducing protein deposition and activation, as well as platelet aggregation. The coating consists of an immobilized noble metal nanostructure in a stochastic distribution. The Bactiguard coating technology, containing Ag instead of Nd, has been clinically proven for other medical device applications mainly in the field of infection prevention^13,16,18,23^. The presented parallel double-blind investigation clearly shows the effectiveness of the novel coating, containing Nd, in reducing acute ST formation, and the methodology presented also provides the opportunity to investigate how in vitro measurements extrapolate to the in vivo situation. The in vitro results show a clear benefit on a number of blood markers, most notably for the TAT complex. TAT generation is a relevant parameter to evaluate the degree of activation of the coagulation system. Protein adsorption was indirectly measured, and a clear effect on protein buildup on the surface was detected, indicating a reduction in fibrin deposition in the coated group. These findings show the coatings potential for reducing the activation of coagulation and thrombus formation. Importantly, the findings were strongly supported by the in vivo data, showing a decrease in thrombus formation compared to paired controls.

Nanostructuring of materials is a common way of achieving superhydrophobic surfaces^36^. Although the investigated coatings do not exhibit such a feature, they show a significant increase in contact angles with DIW, blood plasma, and whole blood (Fig. 5c–h), which could partly explain the decreased protein adsorption. For example, nanostructured titanium has been shown to reduce the immune response, specifically monocyte adhesion, although in vitro^37^. Hydrophobic surfaces have also been shown to increase the flow shear rate at the surface, facilitating easier protein removal after eventual buildup^38^. These effects in combination could explain the reduced levels of TAT and other biomarkers in the coated group compared to controls in the in vitro experiments for both porcine and human blood^39–41^. The present findings corroborate previous studies showing that the absorption of fibrinogen on noble metals is reduced^14^. As mentioned above, this reduction is believed to be directly related to the degree of nanotopography that the metal composition shows in the SEM analyses^42^. In contrast to this, hydrophilic surface properties have been demonstrated to facilitate endothelialization^43^. Arguably, an equally important property is to inhibit protein adhesion that both improves endothelialization and reduces the subsequent thrombus formation cascade reaction^44^. Although the coating in the present study is not hydrophilic it is believed to provide an increased hemocompatibility due to the reduced protein adhesion.

In vitro fibrin deposition in the present investigation (Fig. 3) was also reduced in the coated group. An explanation could be that the control group surface has no mechanism for removing the deposited proteins, resulting in an exponential activation of the thromboembolic system. In the coated groups, however, one effect could be that protein buildup is transported away through the flow by shear force or occur due to a conformational change of adsorbed proteins. The palladium (Pd) coating matrix acts as a catalyst in creating these conformational changes^45,46^. This effect is relevant for the initial protein adhesion and activation of FXII and FXI in the contact activation mechanisms. Furthermore, Nd acts as a calcium inhibitor in the enzymatic reactions of factor X and thrombin^25^. The in vitro results absolute data, as described, cannot be directly extrapolated to an in vivo situation since it is a closed system, amplifying the thrombogenicity of these materials and data illustrating consumption in a closed loop. In an in vivo situation, there is a continuous supply of fresh blood which gives a different situation when interpreting the different parameters. With that said, there are several statistically significant differences between the coating and the control group, as seen in Fig. 3.

This study focuses on the stent material performance in the acute stage since it also creates significant clinical challenges, meaning there is much to be learned about the thrombogenicity over longer time periods with this coating. However, porcine blood is more thrombogenic than the human counterpart and could potentially simulate longer term results in humans when exposed to stent implants^47^. Furthermore, the build-up and composition of the adsorbed protein layer vary greatly over time and are impossible to predict, which warrants further studies also with longer follow-up^48^. In certain clinical scenarios stents are implanted in atherosclerotic lesions, which would behave differently compared to this study. In general, the main factor determining thrombocyte aggregation and thrombus formation in the acute phase is the blood interaction with the foreign material. In the situation of stent implantation in a atherosclerotic artery segment, there is an increased risk of presence of microdissections and thereby exposure of atherosclerotic material and other vascular components to the implanted foreign material. This would increase the interaction with tissue factors and increase thrombocyte activation, but the subsequent mechanism leading to thrombus formation is the same^49–51^.

The feared complication of acute ST could potentially be reduced with this technology, and thereby alleviate the need for DAPT. When discussing the clinical implications of this, also in the longer term after implantation, one has to consider competing stent technologies, such as DES and bioresorbable stents. DES are widely utilized as they have been shown to reduce the rates of both ST and ISR compared to BMS in the long term^9^. Although DES have been substantially developed over the years, there are still concerns over the long term effectiveness due to delayed endothelialization, inflammatory reactions and neoatherosclerosis, leading to an increased risk of late-stage ST^10,52–54^. The bioresorbable stent was intended to solve some of the issues of DES but still suffer issues in terms of mechanical strength and negative effects of degradation products^55^. They also do not focus on the acute stage after implantation and as described above there are several clinical challenges with DAPT in the acute and subacute stage. The standard clinical practice is to give DAPT for 6–12 months, even with newer stent platforms, although this is still up for debate^5^. The coating reported here has the potential to improve the outcome of acute ST, thereby potentially reducing the need for DAPT treatments acutely. Furthermore, we believe this coating could be an improvement compared to competing stent platforms since the coating does not show signs of deterioration over time, but this has to be tested in long-term experiments.

**Figure 6:**
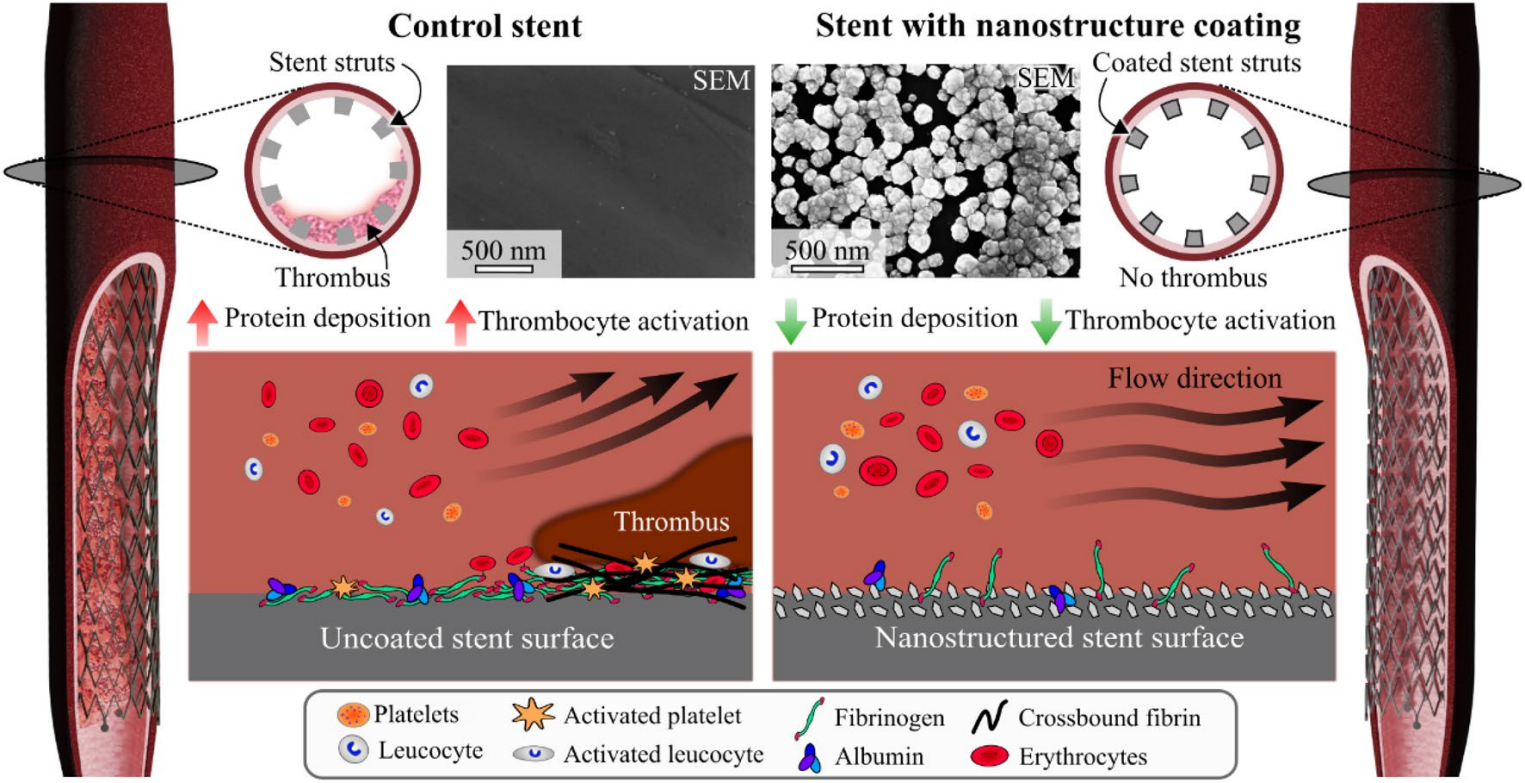
Hypothesis of the observed thrombosis phenomena and how they relate to the studied nanostructure coating. An example of stenosis (left stent). The stent without the coating shows extensive protein build-up, platelet aggregation, and leucocyte activation on the surface. The nanostructured stent (right stent) shows a reduced protein deposition, yielding a lower degree of platelet aggregation, leucocyte activation, and subsequently lowered thrombus formation.

Several measurement modalities were utilized here, with all pointing to an improved response to the coating compared to the control. This demonstrates a benefit of using immobilized noble metal nanostructure coatings on nitinol-based endovascular implants. In vitro results showed a substantial improvement on a number of biomarkers, most notably for the TAT complex, and in vivo results showed a significant difference between the coating and control in terms of acute thrombus formation, in favor of the coating. These effects are influenced by the coating nanotopography, which increases the contact angle with blood and blood plasma, and is believed to induce conformational changes of adsorbed proteins. The hypothesized effect of the coating is a reduction of initial protein adsorption and subsequent reduction of the coagulation cascade through the intrinsic pathway and the inflammatory system (Fig. 6). Future clinical implications of these findings could be a reduced need for DAPT in the acute and subacute phase. Potentially, the need for DAPT could be reduced also in the longer term if additional studies show a lasting effect of the novel coating.

### Supplementary Information

Supplementary Information.

## Data Availability

The datasets used and/or analyzed during the current study available from the corresponding author on reasonable request.
