# Supplementary material for: A novel noble metal stent coating reduces in vitro platelet activation and acute in vivo thrombosis formation: a blinded study
Source: Sci Rep. 2023 Oct 11;13:17225. doi: 10.1038/s41598-023-44364-4 (PMC10567768; doi:10.1038/s41598-023-44364-4)
Supplement: Supplementary file 1 — Supplementary Information. [file 41598_2023_44364_MOESM1_ESM.docx]

**
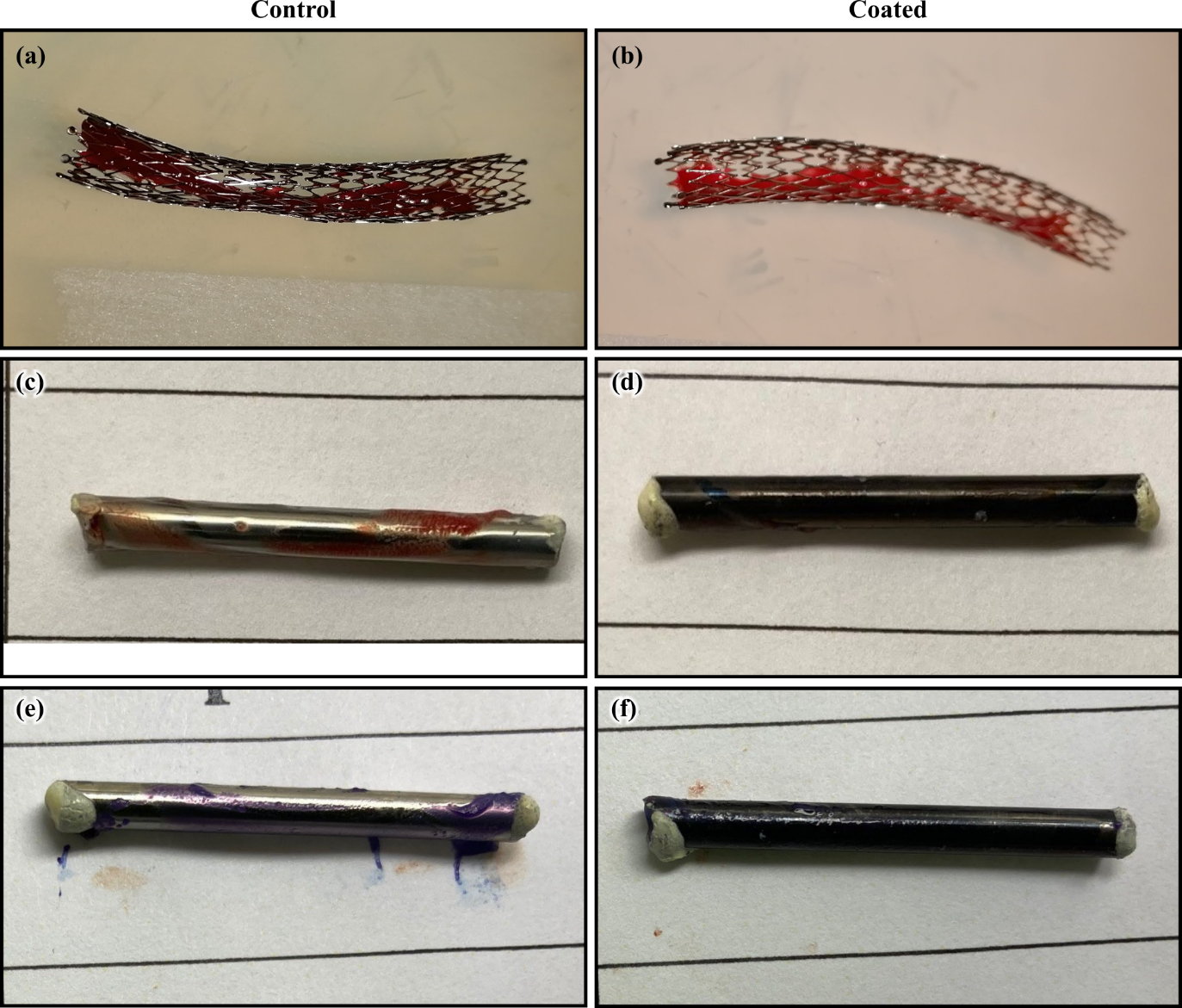
**

Supplementary figure 1. Examples of explanted stents (a-c), nitinol tubes after CHL experiments (d-f) and hematoxylin staining of nitinol tubes (e-f). The fibrin deposition on the control tubes was measured in ImageJ, indicating a significant improvement in the coated groups

Supplementary table 1. Data from in vivo blood samples taken at time points P1-P6. No significant differences were found for any measured parameter. On systemic blood samples, the effect of the interventions during the surgical procedure has a more substantial influence on the measured markers than the coatings' ability to reduce these.

|  | Implant order | P1 | P2 | P3 | P4 | P5 | P6 |
| --- | --- | --- | --- | --- | --- | --- | --- |
| TAT  [μg ml^-1^] | Coating – Control | 6.7 ± 3.5 | 11.1 ± 4.4 | 9.1 ± 3.7 | 10.7 ± 7.2 | 18.7 ± 11.0 | 16.7 ± 13.9 |
|  | Control – Coating | 5.5 ± 1.1 | 6.4 ± 1.4 | 9.3 ± 1.9 | 14.8 ± 6.8 | 15.3 ± 18.5 | 20.1 ± 16.2 |
| B-TG  [IU ml^-1^] | Coating – Control | 2.8 ± 1.5 | 2.1 ± 1.6 | 5.2 ± 3.8 | 2.5 ± 3.0 | 2.0 ± 3.1 | 8.3 ± 6.5 |
|  | Control – Coating | 2.8 ± 1.9 | 2.3 ± 1.6 | 4.5 ± 3.9 | 2.6 ± 1.9 | 3.8 ± 3.7 | 8.1 ± 5.9 |
| PMN  [ng ml^-1^] | Coating – Control | 8.8 ± 2.7 | 8.9 ± 2.8 | 14.5 ± 6.8 | 11.0 ± 4.7 | 11.0 ± 4.2 | 9.9 ± 3.8 |
|  | Control – Coating | 6.8 ± 5.0 | 7.0 ± 2.6 | 9.7 ± 2.9 | 11.8 ± 3.0 | 10.8 ± 4.5 | 12.8 ± 11.2 |
| ADP  [U] | Coating – Control | 88.5 ± 16.1 | 82.3 ± 15.5 | 82.5 ± 7.9 | 76.3 ± 4.9 | 72.3 ± 10.3 | 68.0 ± 2.2 |
|  | Control – Coating | 61.0 ± 28.3 | 65.5 ± 22.5 | 62.3 ± 29.9 | 64.3 ± 19.6 | 60.5 ± 15.0 | 64.8 ± 23.6 |
| TRAP [U] | Coating – Control | 18.0 ± 11.5 | 21.3 ± 8.5 | 14.3 ± 7.4 | 12.8 ± 9.2 | 14.0 ± 4.4 | 8.0 ± 1.0 |
|  | Control – Coating | 42.5 ± 10.8 | 39.0 ± 19.7 | 34.8 ± 8.8 | 34.3 ± 11.0 | 27.5 ± 19.0 | 37.5 ± 18.2 |
